# Biomarkers of dissociation

**DOI:** 10.1192/bjo.2023.511

**Published:** 2023-07-03

**Authors:** Antje A. T. S. Reinders, Allan H. Young, Dick J. Veltman

**Affiliations:** Department of Psychological Medicine, Institute of Psychiatry, Psychology and Neuroscience (IoPPN), King's College London, UK; Department of Psychiatry, Amsterdam University Medical Centers, Vrije Universiteit (VU) Medical Center, VU Amsterdam, The Netherlands

**Keywords:** Dissociative identity disorder, post-traumatic stress disorder, functional neurological disorder, genetics, treatment

## Abstract

Dissociative symptoms present transdiagnostically and are related to poor clinical outcome. Research into the biological correlates of dissociation remains limited. This editorial summarises and discusses papers from this themed series of *BJPsych Open* that contribute to unravelling the biological correlates of dissociative symptomatology with the aim of improving treatment and treatment outcome.

Pathological dissociative symptoms disrupt every area of psychological functioning and range from mild detachment from the immediate surroundings to a failure to integrate one's thoughts, feelings and memories, contributing to a lack of sense of self or identity. Pathological dissociation coincides with altered biology and presents transdiagnostically.^1^ Best known are the dissociative disorders such as dissociative amnesia, dissociative fugue, depersonalisation/derealisation disorder and dissociative identity disorder (DID).^2^ However, dissociative symptoms also occur in other disorders, such as post-traumatic stress disorder (PTSD), depression, borderline personality disorder, obsessive–compulsive disorder, bipolar disorder and schizophrenia^1^. Many assert that dissociative disorders such as DID are fictitious and/or rare.^2,3^ On the contrary, DID, the most severe dissociative disorder, has a trauma-related aetiology^2^ and a lifetime prevalence of approximately 1–1.5%,^2^ similar to intensively researched disorders such as schizophrenia and bipolar disorder.^4^ Nevertheless, research output and funding for DID are vastly lagging behind.

Although interest in trauma-related dissociation has surged recently in both academia^1^ and popular media,^5^ evidence-based psychotherapy and pharmacotherapy for trauma-related dissociative symptoms is lacking, contributing to the personal suffering of individuals and high (financial) burden on services.^6–8^ Pathological dissociation is a negative predictor of treatment response^9^ because severe dissociative symptoms are resistant to standard psychological and pharmacological treatment. Trauma-focused psychotherapies do not offer immediate relief to the individual involved and are time-consuming. Pathological dissociative symptoms are thus related to protracted personal suffering and high direct and indirect societal costs.^8^ The major reason for the absence of tailored (individualised) treatment is that pathological dissociation is under-researched and its underlying (neuro)biological mechanisms remain poorly understood. Therefore, research into the (neuro)biology of dissociation that could lead to the development of faster, targeted psychological or pharmacologically assisted interventions is needed and important.^10^ These reasons led to this themed series in *BJPsych Open* on biomarkers of dissociation, seeking contributions describing original research and systematic reviews, along with editorials, short reports and case studies investigating the neurobiology of dissociation. Our focus was on trauma-related/pathological dissociation, treatment outcome and related biomarkers. However, contributions on dissociation in general, including chemically/pharmacologically induced and normal dissociation, were also considered.

## Themed series: ‘Biomarkers of dissociation’

### Neurostructural correlates of normal and pathological dissociation

Normal, non-pathological dissociation is related to daydreaming and mind-wandering as well as altered affect and autobiographical memory. The contribution by Badura Brack and colleagues^11^ investigated for the first time the neurostructural correlates of normal dissociation in a group of 180 children. They aimed to reveal neurostructural biomarkers of trait dissociation in healthy children while correcting for age, gender and traumatisation. Their results showed increased volume of the precuneus in children with higher levels of trait dissociation, which was not related to antecedent trauma exposure. Because of the pioneering nature of their research the authors discussed their finding in relation to neurofunctional correlates of both normal dissociation, that is mind-wandering, and pathological dissociation. Functional correlates of mind-wandering identified the importance of the precuneus/posterior cingulate cortex during this dissociative state, whereas the functional correlates of pathological dissociation included the posterior association areas. The authors therefore concluded that the precuneus is an essential brain region to consider in future dissociation research.

The precuneus was also implicated in a working memory study included in this themed series in individuals with a DID. Vissia and colleagues^12^ acquired both behavioural data and neural activation patterns in 92 sessions during a working memory task. Genuine diagnosed DID and DID-simulating controls participated as authentic or simulated neutral and trauma-related identity states while a paired control group of individuals with PTSD and healthy controls participated as themselves. Identity state-dependent behavioural performance and neural activation were found, and DID simulators made fewer errors of omission than those with genuine DID. A second study^13^ in this themed series extended this sample of individuals with DID to address inconsistencies in findings regarding amygdala volume in relation to dissociation (see for review^1^). The study did not show differences in amygdala global and subregional volumes between the DID and the healthy control groups. A third study, by Daniels and colleagues,^14^ further challenges the function of the amygdala in dissociative processing of trauma-related memories.

### Trait and state dissociation

A unique study in individuals with a dissociative disorder not otherwise specified (DDNOS) used electroencephalography (EEG) of frontal brain regions to examine neural processing underlying acute dissociation. This study by Schäflein and colleagues^15^ is particularly interesting because it includes measures of both trait and state dissociation. This is important because little is known about how neurobiological processes are related to different dissociative symptoms (see for review^1^). Indeed, the study showed that experimentally elicited acute dissociation in people with DDNOS correlated positively with total EEG power at the beginning of a negative self-relevant condition but that this association was not present for trait dissociation. Despite the notes of caution and limitations of the study, the suggestion of the authors that their finding could serve as a brain biomarker for state dissociation is an important avenue to explore further.

### Null finding in a script-driven imagery study

The involvement of frontal brain regions in pathological dissociation has been one of the most consistent findings transdiagnostically and independent of stimulus paradigm (see for review^1^). However, as highlighted in the paper by Mertens and colleagues,^16^ it remains important to corroborate results in independent samples. Their study used the script-driven imagery paradigm but did not confirm *a priori* hypothesised brain areas despite being one of the largest studies of its kind. Because of the relatively large sample and methodological rigour of the study we thought it important to publish this null finding in this themed series. A comment^17^ on this paper discusses possibilities to enhance neurobiological responses while keeping the script-driven imagery paradigm as the gold standard. One option provided was to integrate responsiveness of other biomarkers, such as physiological markers, with the neurofunctional markers. Analysing these biological responses to the script-driven imagery paradigm conjointly could increase response detection sensitivity. Additionally, we would like to propose implementing event-related designs presenting trauma-related words instead of scripts, which would allow for capturing short, transient neurofunctional responses.

### Systematic reviews and meta-analyses: FND, genetics

This themed series also invited systematic reviews and meta-analyses. A review of dissociation and its biological and clinical associations in functional neurological disorder (FND) by Campbell and colleagues^18^ might be perceived as controversial, because although FND is currently classified as a dissociative disorder in ICD-11, this conceptualisation is not widely accepted. The findings that dissociation in FND is associated with structural and functional brain alterations, including frontal brain regions, are clinically relevant and support the classification as a dissociative disorder.

The most deprived area of research in relation to dissociative symptoms is that of genetics. Fewer than 10 studies have investigated genetic biomarkers of dissociation and, owing to the lack of overlap in methodologies and samples, they are difficult to compare (see for review^1^). The most interesting study to date has been published in this themed series because Lee and colleagues^19^ investigated gene–environment interactions with childhood maltreatment and dissociative symptoms in a trauma-exposed sample with or without a diagnosis of PTSD. Two oxytocin receptor gene single nucleotide polymorphisms (SNPs) were found to be associated with dissociative symptoms but not with a diagnosis of PTSD. A third SNP was found to be associated with dissociative symptoms when interacting with early childhood traumatisation. Such studies encourage future research into the genetics of trauma-related dissociation.

## Paucity of research on biomarkers of dissociation

Current scientific research is aimed at identifying specific biomarkers underlying psychiatric symptoms. Biomarkers can serve as indicators of symptom mechanisms and of response to treatment and are the foundations to a precision medicine approach to (mental) disease. Precision medicine aims for the individualisation of diagnosis and treatment. Of the four biomarker categories of interest, namely neurobiological, psychobiological, psychophysiological and genetic biomarkers (see for review),^1^ we received submissions on only two categories, namely neurobiology and genetics. We did not receive any submissions investigating blood biomarkers of dissociation, including inflammatory markers, or investigating the psychophysiology of dissociation, including blood pressure and heart rate. This was surprising because psychobiological markers such as increased oxytocin and prolactin and decreased tumour necrosis factor alpha (TNF-α) hold a promise of importance for the field. Psychophysiological biomarkers, including blood pressure, heart rate and skin conductance, are under-researched for dissociation. Furthermore, such relatively cheap measures hold the promise of quick diagnostics, targeted pharmaceutical intervention and prediction of treatment response, as found for other disorders, such as depression. Therefore, we urge future research efforts and funding allocations to include such blood and physiology markers of dissociation.

## Conclusions

This themed series aims to contribute to understanding the biology underpinning dissociation. The studies included provide novel information regarding genetics, brain function and/or structure in disorders affected by dissociative symptoms, including DID, DDNOS, PTSD and FND, as well as in healthy people, and further our understanding of the biological correlates of dissociation.

## Data Availability

Data availability is not applicable to this article as no new data were created or analysed in this study.
